# Multi-Objective Optimization of Injection Molding Parameters for Manufacturing Thin-Walled Composite Connector Terminals

**DOI:** 10.3390/ma17163949

**Published:** 2024-08-08

**Authors:** Mingbo Tan, Size Peng, Yingfei Huo, Maojun Li

**Affiliations:** 1 State Key Laboratory of Advanced Design and Manufacturing Technology for Vehicle, Hunan University, Changsha 410082, China; 2 Hunan Xingtu Aerospace and Spacecraft Manufacturing Co., Ltd., Zhuzhou 412000, China; 3 Yangzhou Pinghang Aerodynamics Technology Co., Ltd., Yangzhou 225200, China

**Keywords:** composites, injection molding, multi-objective optimization, flow characteristics

## Abstract

The rapid development of new energy vehicles demands significant improvements in connector structures and performance standards. Wire harness connectors, crucial for linking various electrical components, face challenges due to their small size and thin-walled structure, which can lead to dimensional shrinkage and warping during injection molding. To address these issues, this study optimizes the injection molding process by fine-tuning parameters such as melt temperature, mold temperature, injection time, holding pressure/time, and cooling time. By integrating the Taguchi method with grey relational analysis, the study enhances the molding process for thin-walled composite connectors. This combined approach provides a comprehensive framework for optimizing multiple quality objectives and improving the overall performance of injection-molded composite components.

## 1. Introduction

The rapid evolution of new energy vehicles has escalated the demand for enhanced connector quality. Wire harness connectors, serving as devices to link wires and cables, streamline the electrical connection process, ensuring reliable connections and shielding wires from external environmental factors. Given the small size and intricate hole structures of wire harness connector terminals, there is a heightened need for precise production and manufacturing. Plastic injection molding emerges as a fitting solution for the lightweight design of automotive connectors, adept at handling complex geometric dimensions and structural components [1]. This method offers the advantages of cost-effectiveness and high production efficiency, enabling the creation of intricate structural parts in a single step while achieving high-quality surfaces [2].

Plastic injection molding involves four pivotal stages, including melt filling, pressure holding shrinkage, cooling molding, and demolding. Producing high-quality surface injection-molded parts in a single manufacturing cycle is challenging due to the interplay of factors like molds, materials, and process parameters [3]. Defects such as warping deformation, dimensional shrinkage, and incomplete bonding frequently arise. Traditional approaches relying on empirical and trial-and-error methods heavily depend on operator experience. This reliance makes it challenging to effectively address issues encountered in the production process of intricate injection-molded parts, leading to increased production costs and heightened complexity during the design phase [4]. Therefore, to reduce experimental costs and enhance the injection molding quality, numerical analysis is employed to elucidate the influence of process parameters on injection molding process and the resultant quality of the target part [5].

The porous structure and thin-walled characteristics of wire harness connector terminals make them susceptible to hole size shrinkage and warping deformation. Pachorkar et al. [6] explored the impact of injection-molded part structure on structural molding quality, while Guerra et al. [7] conducted a comprehensive study on the effects of plastic part geometry, packing pressure, and post-treatment conditions on injection-molded part quality. Research indicates a close relationship between warping and factors such as geometric shape, forming process parameters, post-forming conditions, and the relief of residual stress in polymer structures. On the other hand, shrinkage is primarily influenced by the geometric shape of the parts. Simultaneously, there is a growing focus on the influence of process parameters in injection molding. Karagöz [8] studied the effect of mold surface temperature on plastic parts, revealing a 2% increase in warping deformation for every 10 °C rise in surface temperature. Gim et al. [9] found in their research that the mold temperature on the surface glossiness of injection-molded products significantly impacts the final outcome. Wang et al. [10] conducted an in-depth exploration of the differences in cavity pressure between simulation and actual molding, proposing simulation adjustment methods to minimize disparities. This optimization of injection molding simulation analysis significantly enhances prediction accuracy and improves production efficiency in actual molding processes.

The researchers conducted detailed research on the optimization of process parameters. To find the optimal combination of these parameters, minimize the number of experiments, and save costs, the Taguchi method emerged as a suitable choice [11]. Optimizing process parameters can significantly enhance forming quality and reduce production costs [12]. Henti et al. [13] utilized the Taguchi method to design orthogonal experiments and employed signal-to-noise ratio analysis to identify the optimal process parameters. Lin et al. [14] combined the Taguchi method and grey relational analysis to study micro-sized parts, revealing that mold temperature is the most crucial parameter in the powder injection molding process, particularly for mitigating warping phenomena. Bademlioglu et al. [15] optimized process parameters in the organic Rankine cycle using the Taguchi method and grey relational analysis, showcasing the versatility of this approach across different domains of study.

When dealing with the optimization of a single objective, determining a set of solutions allows for finding the best outcome. However, addressing multiple quality objectives presents a challenge as it is not feasible to directly obtain a set of solutions that resolve conflicting quality goals [16]. In the context of handling multi-objective problems, a common approach involves weight allocation to target qualities, followed by the application of intelligent algorithms for multi-objective optimization [17]. Moayedian et al. [18] employed artificial neural networks and Taguchi technology in conjunction with the analytic hierarchy process to assign weights to various defects in thin-walled parts. There is a growing trend towards the continual refinement and enhancement of optimization methodologies [19,20]. For instance, layered sampling and comprehensive entropy weighting methodologies are being integrated to optimize injection molding parameters for thin-walled propeller blades [21]. Additionally, Gao et al. [22] demonstrate the efficacy of utilizing the BO-RFR and NSGA II methods in achieving multi-objective optimization within the domain of injection molding. Feng et al. [23] integrated a hybrid artificial neural network (ANN) with a multi-objective genetic algorithm to address the minimization problem of warping and volume shrinkage defects in injection-molded products.

**Figure 1:**
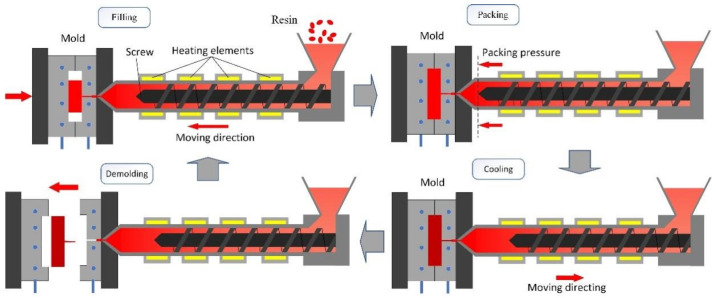
Schematic showing the injection molding process.

The Taguchi method employs an orthogonal array design to optimize process parameters with the aim of enhancing product quality and stability while minimizing experimental trials and costs. Grey relational analysis (GRA), on the other hand, effectively handles uncertainty and multi-objective optimization issues, particularly in scenarios with scarce or low-quality data. By integrating these two methodologies, a comprehensive analysis of the relationships among various factors can be achieved, leading to well-rounded optimization results. Specifically, this approach is applied to the multi-objective optimization of process parameters related to warpage and shrinkage in connector terminals, as showing in the Figure 1. This is further complemented by Moldflow, signal-to-noise ratio analysis, and variance analysis for data processing, ultimately resulting in optimized parameter combinations that minimize warpage and shrinkage. These results could provide valuable insights and references for the manufacturing of composite wire harness connector terminals.

## 2. Methodology

### 2.1. Simulation Model

**Figure 2:**
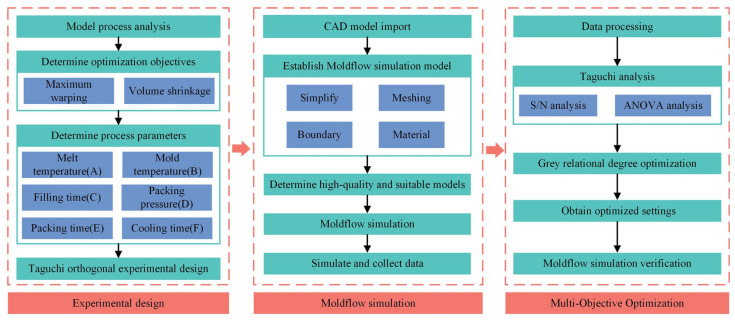
Flowchart of the proposed method.

Figure 2 outlines the approach for structural analysis and parameter optimization of injection-molded parts. The simulation process in plastic injection molding initiates with three-dimensional CAD parts and concludes by recording the volume shrinkage and warpage of the injection-molded components. The sequential steps in this process are as follows:

1. 3D CAD model of injection mold: Identify the research subject and conduct an initial analysis of the dimensional structure and characteristics of the model.
2. Finite element model of injection molding: Creating a finite element model before simulating injection molding involves several steps.
3. Plastic injection molding simulation: Utilize the commercial software Moldflow for conducting injection molding simulations to acquire comprehensive CAE feature data of injection-molded components.
4. Data optimization: Aggregate simulation outcomes pertaining to warpage and volume shrinkage across diverse process parameters. Perform sensitivity analysis on parameters and undertake multi-objective optimization on the collected data.

Warping and shrinkage are prevalent issues frequently encountered in injection molding processes. The primary cause of shrinkage in plastics is the difference in density between the molten state and the cooled, solid state of the polymer. Conversely, warping arises mainly due to disparities in cooling, shrinkage, and orientation effects. Ensuring dimensional accuracy necessitates suitable mold design and optimal process parameter settings. Effective simulation outcomes necessitate appropriate process parameter settings and model simplification to align with engineering reality. The following outlines the key steps in injection molding simulation analysis.

1. Model simplification: Optimizing the 3D CAD model is imperative prior to meshing. Simplification plays a pivotal role in achieving equilibrium between the overall mesh quantity and quality of the model. By eliminating features with minimal impact, the model is optimized to enhance the efficiency of injection molding simulation analysis. This approach ensures the simulation process retains accuracy and depth while circumventing unnecessary computational intricacies associated with features that exert minimal influence on overall quality characteristics [24].
2. Mesh division: The utilization of double-layer meshes for injection molding simulation analysis, particularly applicable to thin-walled components with width-to-thickness ratios surpassing 4, represents a pragmatic and efficacious methodology. Employing double-layer meshes also recognized as shell elements, proves advantageous in augmenting computational efficiency and adhering to the specialized demands of thin-walled components.
3. Boundary conditions: In the injection molding process, the primary consideration revolves around gate positioning and the number of gates. Varied gate positions yield distinct directions and lengths of molten flow, directly impacting stress distribution and shrinkage alterations on the surface of injection-molded parts. Maintaining equilibrium in melt flow aids in averting localized overpressure occurrences and facilitates warping deformation control. At the same time, straightforward parts allow for gate positioning based on experience or precedent cases, complex structured parts pose challenges in effectively pinpointing the optimal gate position.
4. Material characteristics: The Moldflow material library categorizes the accuracy of materials used in filling, pressure holding, and warping analysis through a quality indicator. When dealing with specialized materials, it becomes imperative to reconstruct mathematical models to uphold simulation accuracy.
5. Solver calculation: Upon finalizing the mold structure analysis model, the next step involves utilizing the finite element solver embedded within the commercial software for computation.
6. Simulation analysis results: After completing the simulation calculation, it is imperative to analyze the variations and distribution of warpage and volume shrinkage. Initially, validate the simulation results to identify evident defects such as insufficient filling, weld lines, and air entrapment.

### 2.2. Parameter Optimization

Through plastic injection molding simulation, insights into the warping and volume shrinkage characteristics of injection-molded parts are garnered. Subsequently, a sensitivity analysis of process parameters is undertaken to scrutinize the impact of each parameter on warping and volume shrinkage. The subsequent phase involves designing orthogonal experiments to facilitate multi-objective data optimization. Taguchi orthogonal and grey relational analysis represent effective methodologies for achieving data optimization, enabling the acquisition of optimal process parameters with minimal experimental iterations. The primary objective of multi-objective optimization is to strike an optimal balance among various objectives, maximizing benefits across multiple dimensions and attaining the optimal solution [25,26].

The S/N ratio can indicate the degree of influence that different parameters have on the target quality. There are three main applications of the S/N ratio: nominal-the-best, larger-the-better, and smaller-the-better. For warpage deformation and volumetric shrinkage, a smaller value is preferred. Therefore, this paper employs the smaller-the-better S/N ratio for analysis. The formula for calculating the S/N ratio is as follows:

(1) $$S/N=-10{log}_{10}\left(\frac{1}{n}\sum_{i=1}^{n}y_{i}^{2}\right)$$

where n represents the number of experiments and $y_{i}$ is the measured value of the *i* experiment. The process of grey relational analysis is as follows:

1. Determine the reference sequence and comparison sequence

Initially, the reference sequence and comparison sequence (*k* = 1, 2, 3 … n; *i* = 1, 2, 3 … m) are determined, where k represents the total number of experiments and m represents the total number of observations. Subsequently, data normalization is conducted on the comparison sequence, employing diverse data processing methods tailored to specific requirements. The objective of normalization is to standardize the data, thereby facilitating equitable comparisons. Various techniques, such as Min-Max normalization or Z-score normalization, may be employed based on the characteristics of the data. Following normalization, grey relational coefficients are computed to quantify the relationship between the reference and normalized comparison sequences. These coefficients provide insights into the strength of the relationship, with higher values indicating more robust correlations. The analysis process may involve iterations, allowing for adjustments based on the obtained results and refining the methodology to enhance accuracy.

2. Data standardization

For the quality goal of achieving greater improvements, the normalization method for raw data is outlined as follows:

(2) $$X_{i}^{*}(k)=\frac{X_{i}^{o}(k)-min{.X}_{i}^{o}(k)}{ma{x.X}_{i}^{o}(k)-{min.X}_{i}^{o}(k)}$$

For the quality goal where smaller values are deemed preferable, the normalization method for raw data is as follows:

(3) $$X_{i}^{*}(k)=\frac{ma{x.X}_{i}^{o}(k)-X_{i}^{o}(k)}{ma{x.X}_{i}^{o}(k)-{min.X}_{i}^{o}(k)}$$

For specific target values, the following raw data normalization method is adopted:

(4) $$X_{i}^{*}(k)=1-\frac{\left|X_{i}^{o}(k)-OB\right|}{max.\left\{ma{x.X}_{i}^{o}(k)-OB,OB{min.X}_{i}^{o}(k\right\}}$$

In the formula, $X_{i}^{o}(k)$ represents the initial sequence, $X_{i}^{*}(k)$ represents the normalized sequence, $ma{x.X}_{i}^{o}(k)$ represents the maximum value in the sequence $X_{i}^{o}(k)$, and ${min.X}_{i}^{o}(k)$ represents the minimum value in the sequence.

3. Grey correlation coefficient calculation

The grey relational coefficient can be calculated by normalizing the data using the following formula:

(5) $$\xi (X_{o}^{*}(k){,X}_{i}^{*}(k))=\frac{\Delta_{min}+\lambda \Delta_{max}}{\Delta_{oi}(k)+\lambda \Delta_{max}},\;(0<\xi (X_{o}^{*}(k){,X}_{i}^{*}(k))\leq 1)$$

(6) $$\Delta_{oi}(k)=\left|X_{o}^{*}(k){-X}_{i}^{*}(k)\right|$$

In the presented formula, denoted as (0, 1), the parameter λ assumes a pivotal role in distinguishing coefficients. A smaller λ is associated with heightened discriminatory capability among coefficients. Notably, extant literature widely employs an average λ of 0.5, a choice driven by the pursuit of optimal equilibrium between discerning power and structural stability [27].

4. Grey correlation degree calculation

The grey relational degree is delineated as the amalgamation of weighted grey relational coefficients, formulated as follows:

(7) $$\gamma (X_{o}^{*}{,X}_{i}^{*})=\sum_{k=1}^{n}w_{k}\xi (X_{o}^{*}(k){,X}_{i}^{*}(k))$$

(8) $$\sum_{k=1}^{n}w_{k}=1$$

$\gamma (X_{o}^{*}{,X}_{i}^{*})$ denotes an assessment metric encompassing multiple quality objectives, serving as a yardstick to gauge various factors within the system.

In summary, the plastic injection molding process underwent initial numerical simulation to attain precise values for warping and volume shrinkage. Following this, an orthogonal experimental table was devised utilizing Taguchi’s method and simulated via Moldflow simulation software. Subsequently, optimization of process parameters ensued to acquire the optimal distribution of said parameters. Leveraging the outcomes of parameter optimization, a redesign, and optimization of the mold structure were undertaken.

## 3. Case Study

### 3.1. Injection Molding Simulation

The plastic injection molding process is scrutinized to obtain high-fidelity simulation models and understand the deformation characteristics of injection-molded components. Following this, through process parameter optimization, the objective is to achieve an optimal distribution of parameters aimed at minimizing warping and volume shrinkage. This investigation centers on the prefabricated component model of the 32-core connector terminal within a vehicle, specifically tailored for connectors in new energy vehicles. The three-dimensional structural model of the injection-molded part possesses dimensions of 31.60 mm × 20.08 mm × 8.20 mm with a thickness of 1.0 mm. This injection-molded component demonstrates intricate structural features, comprising multiple apertures, rib structures, and intricate surface details, including microscopic markings and numbers.

**Figure 3:**
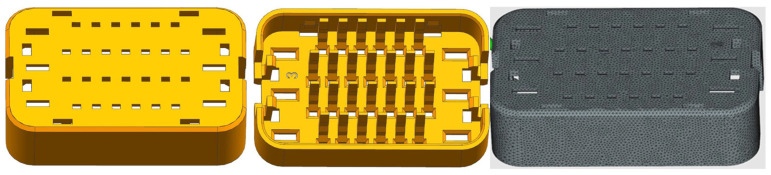
3D structure model and mesh model diagrams.

To optimize the process parameters of injection-molded parts, it is imperative to ensure the development of high-quality injection molding simulation models to uphold the credibility of simulation outcomes. Within the simulation analysis of injection molding, ensuring accuracy primarily hinges on the material, mesh, and parameter settings of the three-dimensional model. Wire harness terminals, being thin-walled parts, are suitable for two-dimensional mesh division. Parameters for triangular mesh division are detailed in Table 1. The maximum aspect ratio of the grid is 19.13, ensuring compatibility with Moldflow’s double-layer grid analysis, with a shape-matching standard surpassing 90%. Figure 3 illustrates the model post-grid partitioning. The material utilized for the terminals in this simulation analysis is manufactured by Celanese (Frianyl A3 GF20 V0 BK 9005/B), boasting a “gold” quality index in the Moldflow material library with the properties presented in Table 2, thereby fulfilling the requisites of the injection molding simulation system.

**Figure 4:**
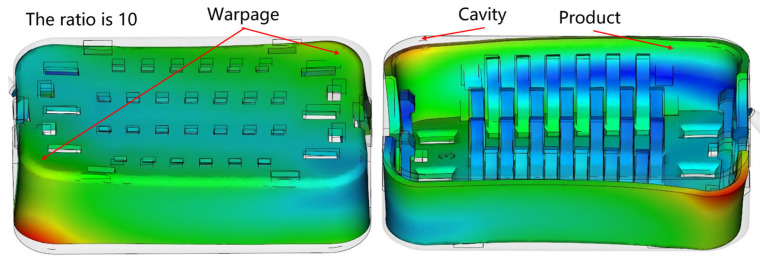
Distribution map of warping deformation.

The analysis model is illustrated in Figure 4. An initial simulation was performed on the injection molding model using the automatic default parameters for the initial mold simulation. The results indicate substantial deformation, primarily along the diagonal axis. Concurrently, an examination of the deformation trend in injection-molded parts was conducted, considering variations in both the cavity and product area. Notably, deformation is relatively minimal at the central region of the model, gradually intensifying as one moves away from the center.

### 3.2. Taguchi Orthogonal Arrays

The Taguchi method, utilized as an experimental design approach for quality and performance optimization, offers notable advantages by extracting comprehensive information through a limited number of experimental runs. This method streamlines the analysis of various parameters, especially in injection molding, resulting in shortened mold design cycles, reduced instances of mold trials, and overall decreased production costs. In this study, five key parameters were selected, including melt temperature (A: Melt_T), mold temperature (B: Mold_T), injection time (C: Filling_t), holding time (D: Holding_P), holding pressure (E: Holding_t), and cooling time (F: Cooling_t), with details showing in Table 3. Employing the Taguchi method, an experimental table L27 (6 × 3) was generated, as outlined in Table 4.

## 4. Results and Discussion

### 4.1. ANOVA Analysis

**Figure 5:**
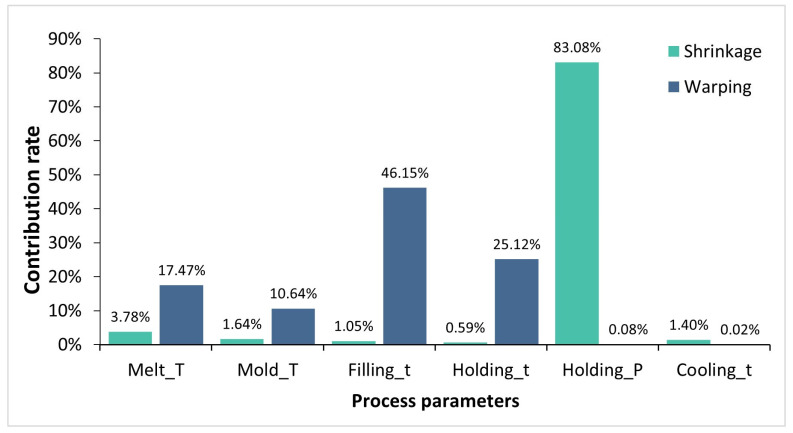
Distribution of contribution for various process parameters.

To quantitatively assess the influence of various process design parameters on quality objectives, specifically warping and volume shrinkage, it conducted a single-objective analysis of variance and calculated the contribution of each factor, with results presented in Table 5 and Table 6 and Figure 5. For volume shrinkage, the *p*-values associated with filling time and holding time are below 0.05, signifying a significant impact on volume shrinkage, with contribution rates of 20.43% and 66.32%, respectively. In the injection molding process, holding pressure analysis aims to supplement the volume shrinkage of the melt during cooling, thereby maintaining pressure equilibrium across various points in the mold cavity, with holding pressure and holding time serving as key parameters. Given the small size of the injection-molded parts, a shorter duration is required for injection and pressure holding. Consequently, injection-molded part quality exhibits high sensitivity to injection and holding times, with holding time exerting the most substantial impact on volume contraction. In the case of warping deformation, the *p*-values associated with melt temperature (A), mold temperature (B), filling time (C), and holding pressure (D) are all below 0.05, indicating a significant impact on warping deformation. These results underscore the dominant role of melt temperature in these factors for small molds of porous wire harness connectors.

### 4.2. The Influence of Process Parameters on Shrinkage

**Figure 6:**
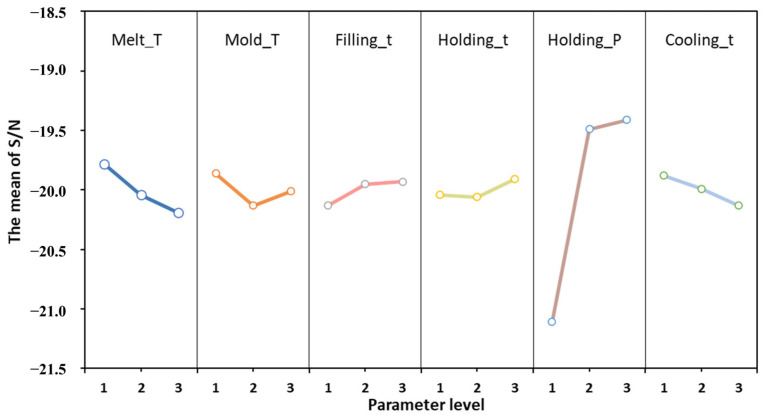
The main effect diagram of S/N for volume shrinkage.

Shrinkage is an unavoidable phenomenon in injection molding, while through the adjustment of processing parameters, it can be effectively controlled and minimized. This ensures the dimensional accuracy, shape integrity, surface quality, and mechanical properties of the molded parts. Figure 6 shows the main effects plot for process parameters based on signal-to-noise ratio analysis. The signal-to-noise ratio serves as a metric to gauge the impact of various parameters on the quality of the target, typically employed through three application methods: visual characteristics, larger-the-better, and smaller-the-better. In the context of warping deformation and volume shrinkage, where smaller values are preferred, the signal-to-noise ratio with the desired smaller characteristic is utilized for analysis.

Based on the graph’s trend, there is a notable difference between mold temperature and other process parameter changes. Typically, mold temperature positively correlates with the shrinkage rate. As the temperature rises, the shrinkage rate increases. However, as illustrated in Figure 6, the shrinkage rate initially increases and then decreases with rising mold temperature. This phenomenon can be ascribed to the structural intricacies inherent within the components. The focal point of this experimental inquiry rests upon a diminutive, intricate, and porous, thin-walled component. Such components possess a complex architecture predisposed to non-uniform cooling. Even when the mold temperature adheres to recommended thresholds, an irregular cooling system may provoke localized over-cooling, thereby impinging upon the uniformity of shrinkage. Consequently, localized shrinkage amplifies, engendering an abatement in overall shrinkage magnitude. Moreover, owing to the petite proportions of the components, the injection filling duration is abbreviated while the injection velocity is heightened. Concurrently, the elevated mold temperature precipitates a decrement in plastic viscosity. This confluence of factors incites an expedited plastic flow during filling, culminating in an overzealous filling or excessive inundation. The resultant effect is an undue extension, thereby attenuating the shrinkage rate. Concerning ancillary process parameters, the melt temperature and cooling duration exhibit a direct correlation with the shrinkage rate, and the augmentation of these parameters invariably augments the shrinkage rate. Conversely, parameters such as filling duration, holding pressure, and retention time evince an inverse relationship with the shrinkage rate; escalation of these parameters invariably diminishes the shrinkage rate.

The main reason for shrinkage is mainly due to the density difference between the molten and solid states of the polymer at the microscopic level. During the cooling process, the molecular chains change from loose arrangement to tight arrangement, resulting in a decrease in free volume and overall volume contraction. Macroscopically, shrinkage is influenced by factors such as material properties, mold design, processing conditions, and the geometric shape of the workpiece. For example, parameters such as cooling rate, injection pressure, and mold temperature can all affect the final degree of shrinkage.

**Figure 7:**
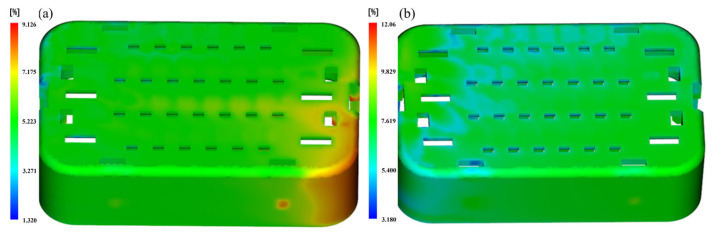
Shrink in the (**a**) optimum and (**b**) worst case.

The above figure shows that the holding time has a significant effect on volume shrinkage, while other process parameters have a relatively small impact on volume shrinkage. In the process of injection molding, the purpose of holding pressure analysis is to supplement the volume shrinkage of the melt during the cooling process and ultimately maintain the pressure balance of various points in the mold cavity, with key parameters of holding pressure and holding time. Due to the small size of the injection-molded parts themselves, less time is required for injection and pressure holding. Therefore, the quality of injection-molded parts has a high sensitivity to injection time and holding time. So, the holding time has the greatest impact on volume contraction. The best combination of process parameters is A1B1C3D3E3F1, with an average volume shrinkage rate of 5.2%, which is the lowest among all experimental tables. At the same time, the combination of process parameters is not included in Table 4. The worst combination is A3B2C1D2E1F3, with the simulation model of this case shown in Figure 7. At this time, the average volume shrinkage rate is 7.6%, which is higher than other experimental results.

### 4.3. The Influence of Process Parameters on Warping

Warping predominantly results from non-uniform cooling across different regions of the workpiece, which leads to uneven internal stress distribution. The discrepancy in shrinkage between areas undergoing rapid cooling and those experiencing slower cooling rates generates internal stresses. Upon demolding, these stresses are released, causing the workpiece to exhibit warpage.

**Figure 8:**
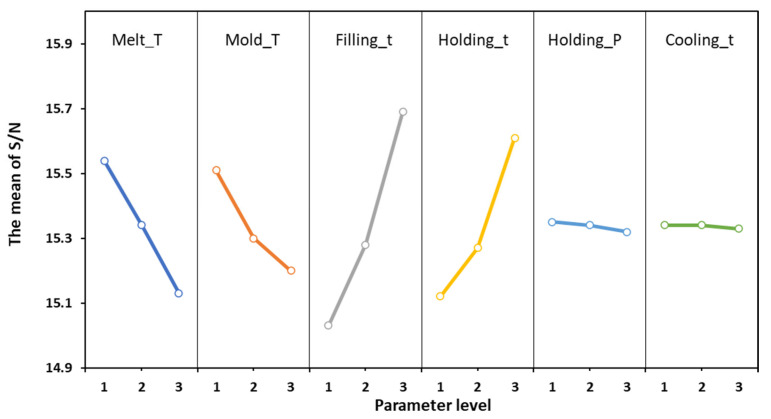
The main effect diagram of S/N for warping deformation.

**Figure 9:**
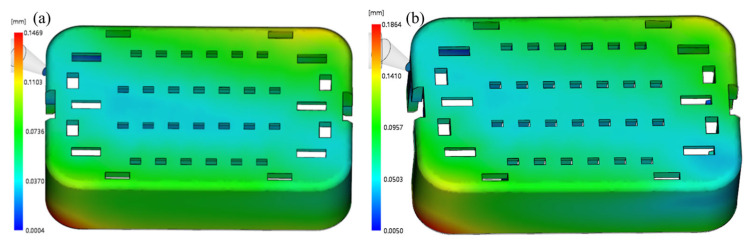
Warping in the (**a**) optimum and (**b**) worst case.

Figure 8 illustrates that among the five injection molding process parameters, filling time exerts the most significant influence. Conversely, cooling time and holding time exhibit minimal impact on the warping deformation of injection molding. A higher signal-to-noise ratio indicates superior performance of process parameters at optimal levels for the target. Consequently, the parameter combination A1B1C3D3E1F1 (melt temperature of 268 °C, mold temperature of 60 °C, filling time of 0.6 s, holding pressure of 85 MPa, holding time of 0.5 s, cooling time of 30 s) is identified as the optimal design value affecting warping in the experiment. The results suggest that the optimal parameter combination can potentially be identified through Taguchi orthogonal experimental design, reducing the necessity to analyze all outcomes. Moreover, as shown in Figure 9, the graph facilitates the identification of the worst parameter combination, with a maximum warpage of 0.1884 mm observed for A3B3C1D1E3F3. Despite variations in process parameters, the warpage behavior of injection-molded parts appears to remain consistent across both scenarios.

### 4.4. Grey Relational Analysis

Grey relational analysis for multi-objective optimization enables the processing of data characterized by non-linear and fuzzy relationships. The primary approach involves transforming multiple objectives into a single objective for data optimization analysis by assigning weighted values to each objective. When performing grey relational analysis, the initial step involves normalizing the data and converting the experimental data into a range between 0 and 1 using mathematical formulas. This normalization process is essential because different quality objectives are often measured in different units, making direct comparisons and optimizations challenging. Normalization is, therefore, necessary to standardize the data into dimensionless forms, facilitating meaningful comparisons and analyses. For quality objectives such as warping and volume shrinkage, where smaller values are preferable, data normalization can be achieved through mathematical formulas. This process ensures that the data are standardized and transformed into dimensionless forms, facilitating effective analysis and comparison. This analysis only has two quality objectives, with a reference sequence of 1.

Table 7 provides the calculation data for the gray relational coefficient and gray relational degree. Regarding the gray relational degree, a larger value indicates a stronger correlation with quality characteristics, thereby suggesting a more optimal combination of process parameters and reduced warping and volume shrinkage deformation. Analysis of the data reveals that the maximum gray relational degree between experiments 8 and 9 is 0.827, signifying the optimal performance of multi-response features under this specific combination of process parameters. These findings aid in identifying the best combination of process parameters to minimize part deformation and volume shrinkage, consequently enhancing product quality.

Analysis of variance was conducted on grey relational data to elucidate the comprehensive impact of various parameters on warping and shrinkage. According to Table 8, the significance of factors ABCDE, as indicated by their *p*-values being less than 0.05, underscores their strong correlation with overall performance. Notably, filling time emerges as the predominant influence, contributing 48.17% to warping deformation and volume shrinkage in connector parts, while cooling time exhibits minimal influence on wiring harness terminals. This phenomenon is attributed to the inherent structural characteristics of the parts. Small and medium-sized components in injection molding demonstrate heightened sensitivity to time, predisposing them to issues such as inadequate filling or localized over-pressure. Additionally, owing to their diminutive size and thin-walled nature, these parts necessitate brief filling and holding times, with the cooling duration set to effectively dissipate heat post-molding and mitigate residual stress impact on the injection-molded components.

**Figure 10:**
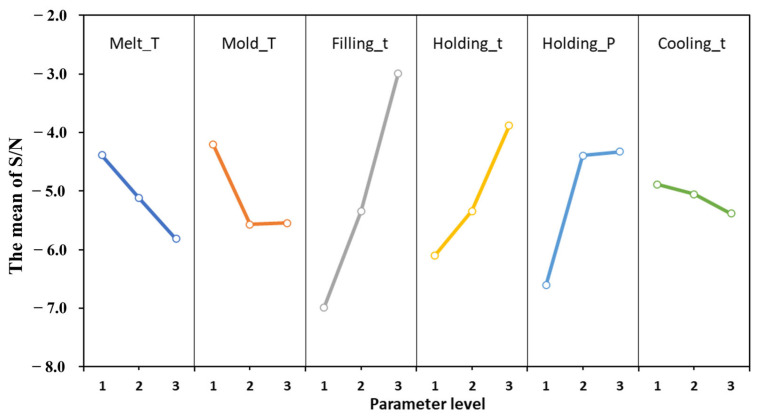
Main effects plot for GRG means.

Based on the main effect diagram of the correlation degree, the optimal combination of process parameters for comprehensive performance characteristics is identified as A1B1C3D3F3F1, as depicted in Figure 10. Through gray relational analysis, it was determined that the optimal parameter combination aligns with the shrinkage optimization parameters. In the optimization process of porous, thin-walled small parts, the consistent distribution of volume shrinkage significantly influences the quality of injection-molded parts. This is attributed to warping deformation, serving as a quality indicator under the comprehensive influence of multiple factors. It describes the deviation degree between the size of the injection-molded part and the cavity size, where the shrinkage difference plays a crucial role in affecting warping.

## 5. Conclusions

To achieve high quality, high productivity, and low energy consumption, this study utilized numerical simulation methods to analyze the effects of process parameters on the shrinkage and warpage of connector terminals. Using Taguchi’s method, along with signal-to-noise ratio and variance analysis, experimental design and data analysis were conducted to determine that filling time has the greatest impact on warpage, while holding time most significantly affects volumetric shrinkage. The results indicated that using the process parameter combination A1B1C3D3E1F1 achieved a minimum warpage of 0.147 mm. The process parameter combination A1B1C3D3E3F1 resulted in a minimum volumetric shrinkage of 5.2%. Grey relational analysis was employed to comprehensively evaluate the molding quality of the connectors,. Variance analysis of the grey relational grade highlighted that filling time has the primary influence on the quality of injection-molded parts. The findings demonstrate that the process parameter combination A1B1C3D3E1F1 yields the highest overall molding quality, with particular emphasis on the impact of shrinkage on porous, thin-walled components. Based on our findings, we propose the following avenues for future research, including exploring the molding process of various porous components to understand their specific behaviors better, examining state-of-the-art simulation technologies, and their integration with real-time monitoring systems to enhance the robustness and accuracy of the molding process.

## Figures and Tables

**Table 1:** Double layer mesh parameters.

Type	Number	Side Length	Aspect Ratio	Matching
Triangle	49,966	0.4 mm	Max: 19.13 Min: 1.16 Average: 1.61	Matching: 92.8% Mutual: 95.3

**Table 2:** Properties of materials used for fabricating connector.

Material Data	Value
Melt/Solid Density [cm^3^]	1.34 g/1.16
Poisson’s ratio v12/v23	0.384/0.52
Shear modulus [MPa]	1947
Average modulus of elasticity [MPa]	5877.29
Tensile modulus [MPa]	7900
Recommended melt temperature [°C]	265–290
Recommended mold temperature [°C]	60–80

**Table 3:** Variable factors and levels selected for analysis.

Levels	A Melt_T	B Mold_T	C Filling_t	D Holding_P	E Holding_t	F Cooling_t
1	268 °C	60 °C	0.2 s	60 Mpa	0.5 s	30 s
2	278 °C	70 °C	0.4 s	70 Mpa	1 s	40 s
3	288 °C	80 °C	0.6 s	80 Mpa	1.5 s	50 s

**Table 4:** Taguchi orthogonal array for injection molding process.

Runs	Factors						Runs	Factors					
	A	B	C	D	E	F		A	B	C	D	E	F
1	1	1	1	1	1	1	15	2	2	3	1	3	1
2	1	1	1	1	2	2	16	2	3	1	2	1	2
3	1	1	1	1	3	3	17	2	3	1	2	2	3
4	1	2	2	2	1	1	18	2	3	1	2	3	1
5	1	2	2	2	2	2	19	3	1	3	2	1	3
6	1	2	2	2	3	3	20	3	1	3	2	2	1
7	1	3	3	3	1	1	21	3	1	3	2	3	2
8	1	3	3	3	2	2	22	3	2	1	3	1	3
9	1	3	3	3	3	3	23	3	2	1	3	2	1
10	2	1	2	3	1	2	24	3	2	1	3	3	2
11	2	1	2	3	2	3	25	3	3	2	1	1	3
12	2	1	2	3	3	1	26	3	3	2	1	2	1
13	2	2	3	1	1	2	27	3	3	2	1	3	2
14	2	2	3	1	2	3	-	-	-	-	-	-	-

**Table 5:** The analysis of variance for volume contraction.

Source	f	Seq SS	Adj SS	Adj MS	F	*p*	Contribution/%
A	2	0.0941	0.0941	0.0471	1.17	0.339	0.85
B	2	0.5261	0.5261	0.2631	6.54	0.01	4.74
C	2	2.2688	2.2688	1.1344	28.21	0	20.43
D	2	0.1114	0.1114	0.0557	1.39	0.282	1.00
E	2	7.3650	7.3650	3.6825	91.59	0	66.32
F	2	0.1763	0.1763	0.0882	2.19	0.148	1.59
Error	14	0.5629	0.5629	0.0402			5.07
Total	26	11.1047					100.00

**Table 6:** The Analysis of variance for warping.

Source	f	Seq SS	Adj SS	Adj MS	F	*p*	Contribution/%
A	2	0.0003010	0.0003010	0.0001505	264.91	0	17.56
B	2	0.0001985	0.0001985	0.0000992	174.68	0	11.58
C	2	0.0007838	0.0007838	0.0003919	689.82	0	45.73
D	2	0.0004210	0.0004210	0.0002105	370.51	0	24.56
E	2	0.0000015	0.0000015	0.0000007	1.29	0.306	0.09
F	2	0.0000003	0.0000003	0.0000001	0.26	0.773	0.02
Error	14	0.0000080	0.0000080	0.0000006			0.47
Total	26	0.0017141					100.00

**Table 7:** Optimal combination provided by Taguchi method.

Runs	GRC		GRG	Runs	GRC		GRG
	Warpage	Shrinkage			Warpage	Shrinkage	
1	0.44	0.46	0.45	15	0.53	0.77	0.65
2	0.45	0.56	0.51	16	0.53	0.77	0.65
3	0.45	0.53	0.49	17	0.37	0.34	0.35
4	0.52	0.44	0.48	18	0.35	0.54	0.45
5	0.51	0.64	0.58	19	0.35	0.55	0.45
6	0.51	0.64	0.58	20	0.62	0.47	0.55
7	1.00	0.48	0.74	21	0.60	1.00	0.80
8	0.94	0.86	0.90	22	0.60	1.00	0.80
9	0.94	0.86	0.90	23	0.39	0.33	0.36
10	0.68	0.43	0.56	24	0.43	0.53	0.48
11	0.69	0.84	0.77	25	0.40	0.65	0.53
12	0.69	0.84	0.76	26	0.34	0.33	0.34
13	0.55	0.48	0.51	27	0.33	0.61	0.47
14	0.44	0.46	0.45	-	-	-	-

**Table 8:** ANOVA result of gray relational grade.

Source	f	Seq SS	Adj SS	Adj MS	F	*p*	Contribution %
A	2	0.0326	0.0326	0.01631	10.18	0.002	4.75
B	2	0.0457	0.0457	0.02285	14.25	0	6.65
C	2	0.3308	0.3308	0.16539	103.18	0	48.17
D	2	0.1151	0.1151	0.05755	35.9	0	16.76
E	2	0.1376	0.1376	0.06879	42.92	0	20.03
F	2	0.0025	0.0025	0.00125	0.78	0.477	0.36
Error	14	0.0224	0.0224	0.00160	-	-	3.27
Total	26	0.6867	-	-	-	-	100.00

## Data Availability

The original contributions presented in the study are included in the article, further inquiries can be directed to the corresponding author.
